# The costs of improving health emergency preparedness: A systematic review and analysis of multi-country studies

**DOI:** 10.1016/j.eclinm.2021.101269

**Published:** 2022-01-27

**Authors:** Lorcan Clarke, Edith Patouillard, Andrew J. Mirelman, Zheng Jie Marc Ho, Tessa Tan-Torres Edejer, Nirmal Kandel

**Affiliations:** World Health Organization, Geneva, Switzerland

**Keywords:** Costs, Health security preparedness, Pandemic

## Abstract

**Background:**

Investing in health emergency preparedness is critical to the safety, welfare and stability of communities and countries worldwide. Despite the global push to increase investments, questions remain around how much should be spent and what to focus on. We conducted a systematic review and analysis of studies that costed improvements to health emergency preparedness to help to answer these questions.

**Methods:**

We searched for studies that estimated the costs of improving health emergency preparedness and that were published between 1 January 2000 and 14 May 2021, using PubMed, Web of Science, Google Scholar, EconLit, and National Health Service Economic Evaluation Databases (PROSPERO CRD42021254428). We also searched grey literature repositories and contacted subject experts. We included studies that estimated the costs of improving preparedness at the global level and/or at the national level across at least ten countries, covered two or more technical areas in the WHO Benchmarks for International Health Regulations (IHR) Capacities, and included activities focused on human health. We mapped costs across technical areas in the WHO Benchmarks for IHR Capacities.

**Findings:**

Ten studies met our inclusion criteria. Costing methods varied substantially across included studies and cost estimates ranged from US$1·6 billion per year to improve capacities across 139 low- and middle-income countries (LMICs) to US$43 billion per year to support national-level activities worldwide and implement global-level initiatives, such as research and development for health technologies (diagnostics, therapeutics, and vaccines). Two recent studies estimated costs by drawing on IHR Monitoring and Evaluation Framework country capacity data, with one study estimating costs across 67 LMICs of US$15·4 billion per year (US$29·1 billion including upfront capital costs) and the other calculating costs for the 196 States Parties to the IHR of US$24·8 billion per year. Differences in included studies’ methods, and the characteristics of countries considered, mean it is difficult to make like–for–like comparisons of the absolute costs or per-capita costs estimated by studies.

**Interpretation:**

Improving health emergency preparedness worldwide will require substantial and sustained increases in investments. Further guidance on estimating the size of those investments can help to standardise methods, allowing greater interpretation and comparison across studies/countries. As well as greater transparency and detail in the reporting of methods by studies focused on this topic, this can help support estimates of global resource requirements and facilitate investments towards improving preparedness for future pandemics.

**Funding:**

None.

**Research in Context**

**Evidence before this study**

As of June 2021, based on searches of electronic databases using search terms “pandemic” OR “epidemic” OR “health emergency” AND “prevention” OR “preparedness” AND “cost”, we found no previous systematic review focused on global or multi-country health emergency preparedness costing studies. Existing research related to this work included reviews of cost-effectiveness studies of specific interventions that contribute to health emergency preparedness, as well as individual articles and reports offering brief summaries of a small selection of studies.

**Added value of this study**

This systematic review offers an assessment of multi-country studies of the costs of improving health emergency preparedness. We synthesise and assess findings from ten included studies, that covered a range of contributory activities and considered a variety of countries. We include a summary of published cost estimates, methods used, and cost drivers. We assess the distribution of activity costs across the categories offered by the WHO Benchmarks for IHR Capacities. We also discuss how the scope of activities considered and costing methods varied across studies.

**Implications of all the available evidence**

Estimates of the costs of improving health security preparedness, through national- and global-level activities, vary across included studies. Both in terms of the calculation methods and the preparedness activities and countries considered. There is a need for more consistent and routinely applied approaches to estimate the costs of improved health emergency preparedness; this could help to improve budgeting and financing for these activities.

Alt-text: Unlabelled box

## Introduction

Over the past two decades, many health emergencies have shown why preparedness is essential and valuable.^1^ Effective preparedness capacities can help communities and countries avoid and minimise the impacts of epidemics and pandemics. All through better capabilities to prevent, detect, respond to, and recover from such disease outbreaks. Simply put, investments in preparedness – facilitated by sizeable, consistent, and predictable public sector-led spending – are essential to improving and protecting people's health, community well-being, and economies.

Despite the health and economic case for investing in health security through epidemic and pandemic preparedness, gaps in financial and political support persist.^2^ Dozens of countries have developed plans, such as National Action Plans for Health Security (NAPHS), to address gaps in preparedness capacities. Yet, achieving full financing for these activities has proven challenging.^3^^,^^4^ Now, with epidemic and pandemic risks at the centre of the global policy agenda, countries have indicated their support for more predictable and sustainable investments that combat emerging pandemic threats and create enduring financing mechanisms.^1^^,^^2^

This systematic review focuses on the costs of improving preparedness, a key part of efforts to develop targets for national- and global-level investments. Several national- and global-level initiatives have emphasised the importance of more funding for preparedness and key next steps include specifying what to spend the money on.^1^^,^^5^^,^^6^ This review's objective was to assess existing evidence, examine methods, and identify cost drivers from available studies.

## Methods

This systematic review follows the recommendations in the Preferred Reporting Items for Systematic reviews and Meta-Analyses (PRISMA) 2020 statement and guidance on conducting and reporting systematic reviews of economic studies from the Cochrane Handbook for Systematic Reviews of Interventions.^7^^,^^8^ See appendix for definitions of key search terms.

### Search strategy and selection criteria

We searched PubMed, Web of Science, Google Scholar, EconLit, and National Health Service Economic Evaluation Databases for studies published between 1 January 2000 and 14 May 2021 (PROSPERO Protocol CRD42021254428). The time period accounted for all studies following the publication of “Influenza pandemic preparedness plan: the role of WHO and guidelines for national and regional planning” in 1999. This document highlighted key issues and questions including “What overall costs are expected from a pandemic, and to enable different responses to it, including enhanced surveillance, monitoring, procurement of materials, etc.?” and predates the International Health Regulations (2005) coming into force in 2007.^9^^,^^10^

We hand-searched publication repositories (Prevention Web, World Bank Open Knowledge Repository), websites, and contacted subject experts to identify additional studies. We conducted a ‘snowball’ search using included studies and other key publications, such as reviews or studies focused on financing issues or the costs of activities in a single country. If necessary, we contacted authors of studies for additional information.

Our search terms were informed by appraising a sample of relevant studies and were refined with the support of librarians. Key terms, which were tailored to each database, included: pandemic, epidemic, health emergency, health security, preparedness, mitigation, cost, and costing. See appendix for further details.

We included all studies that estimated the costs of improving emergency preparedness at the global level and/or at the national level across at least ten countries. This meant that eligible studies estimated, based on market-traded inputs with observable market prices, the costs of improving preparedness to minimise the risks and potential impacts of health emergencies. To be included, studies’ costed activities also had to cover two or more of the 18 technical areas in the World Health Organization (WHO) Benchmarks for International Health Regulations (IHR) Capacities.^11^

Initially, we considered including studies that focused on at least two or more countries and at least one technical area. However, we concluded that included studies needed to account for multiple technical areas and several country settings (with the ten country minimum chosen based on the smallest WHO region containing 11 countries). In our discussion section, we note examples of studies which did not meet our final inclusion criteria and elaborate on this update to our protocol in our appendix file.

We included studies published as grey literature and in peer-reviewed academic journals. We used English language search terms, but did not place restrictions on study languages nor on study design. This meant studies such as economic evaluations met inclusion criteria, as long as they reported aggregate level costs. We excluded studies that focused only on animal health or on activities that occur during the response or recovery phases of health emergencies. See appendix for more on our eligibility criteria.

Our criteria ensured that included studies considered activities across a range of preparedness areas and resource needs across many countries. The 18 technical areas in the WHO Benchmarks for International Health Regulations (IHR) Capacities (“Benchmark areas”) were: 1) National legislation, policy, and financing, 2) IHR coordination, communication and advocacy and reporting, 3) Antimicrobial resistance (AMR), 4) Zoonotic disease, 5) Food safety, 6) Immunization, 7) National laboratory system, 8) Biosafety and biosecurity, 9) Surveillance, 10) Human Resources, 11) Emergency preparedness, 12) Emergency response operations, 13) Linking public health and security authorities, 14) Medical countermeasures and personnel deployment, 15) Risk communication, 16) Points of entry, 17) Chemical events, and 18) Radiation emergencies. See appendix for full descriptions.

All authors developed the search strategy. LC implemented the search and imported study records into Rayyan software.^12^ LC and EP independently screened titles and abstracts of all study records to identify the inclusion candidates. LC and AM then conducted full-text assessments of those candidates. Any disagreements on study inclusion were resolved by consensus and, if required, the inputs of a third author.

### Data collection

We collected data using a standard form in Microsoft Excel. Its design drew on the Consolidated Health Economic Evaluation Reporting Standards (CHEERS) statement and a previous review of the costs and benefits of interventions aimed at major infectious disease threats.^13^^,^^14^ We extracted data for all costed activities; outcomes of interest included the scope and type of costed preparedness activities, the costing methods, estimated costs, and cost drivers. LC and EP independently extracted data for studies and then cross-validated the data extraction. See appendix for further information.

### Data analysis

We collated information about included studies’ characteristics and methods for narrative summary and adapted cost estimates for comparison and analysis. To estimate annual costs when unreported, we divided the total costs estimated in a study, including both capital and recurrent costs, by the number of years costed. In this context, capital costs are fixed/one-time expenditures such as constructing buildings, purchasing equipment, or initial training of staff. Recurrent costs include those following the initial set up of an activity, such as workers’ wages or items used when conducting activities (e.g. diagnostic tests).

To estimate annual per capita costs, we divided annual costs by the total population of countries assessed by the study. If a study did not report this figure, we used estimates of the populations of included countries, at the time of study publication, from the World Bank.^15^ If a study only reported per capita costs, we estimated total costs by multiplying per capita costs by the total population of countries examined by the study.

Initially, we planned to convert all cost data from before 2020 to constant United States Dollars (US$) 2020 by using gross domestic product (GDP) implicit price deflators from the International Monetary Fund (IMF).^16^^,^^17^ However, due to two studies reporting their estimates in US$ 2021, we have used the available first quarter (Q1) 2021 seasonal figures to account for the most recent available estimates.^18^^,^^19^ The base years and currencies for cost data were obtained from studies. If a study did not report the base year for their cost estimates, we used its year of publication.

We examined the distribution of national-level and global-level costs across studies. This involved mapping activities as being “national” or “global”, based on studies’ descriptions and our interpretation during data extraction. We considered costs to be global if they were to, as defined by the 2013 Lancet Commission on Investing in Health, support “global functions” that go beyond the boundaries of individual nations to address transnational issues. These functions include the provision of global public goods (e.g. research and development (R&D) on new tools to tackle neglected diseases), management of negative regional and global cross-border externalities (e.g. outbreak preparedness), and fostering of global health leadership and stewardship (e.g. convening for negotiation).^20^ We acknowledge that national and global health security are closely intertwined and that national capacities are critical to effective global functions, and vice-versa.

In our results, we present the distributions of national-level costs across the World Bank's four income groups—low, lower-middle, upper-middle, and high-income countries—based on the findings reported by studies. We also present summaries of the distribution of capital and recurrent costs, as well as factors noted by included studies that were driving preparedness costs, according to study reporting.

We planned not to conduct assessments of bias using a risk of bias tool (e.g. Cochrane Risk of Bias Tool), acknowledging the already known variability in approaches and that we were evaluating studies' estimated market costs and we were not including estimates of the consequences of interventions. Instead, our conclusions were informed by summarising and comparing the different methods used by studies, but not questioning their validity. We also acknowledge the conceptual nature of this topic area could mean significant variance in studies' approaches to describing activities and estimating costs.

To analyse what types of activities were included in studies, we used each study's descriptions of methods and results to map all costed activities to one or more of the 18 areas of the WHO Benchmarks for IHR Capacities.^11^ LC and EP conducted the initial mapping during data collection. NK and MH, experts in the implementation of preparedness capacities at national and global levels, then reviewed and updated the mapping of costed activities. We then summarised the presence of each Benchmark area in included studies and the number of times each Benchmark area appeared across all of a study's costed activities.

This mapping informed an exploratory analysis of the distribution of studies' costs across the 18 Benchmark areas. We used two separate approaches. The first approach assumed that if a costed activity mapped to two or more Benchmark areas, its costs were evenly distributed across each area. For example, if an activity mapped to four Benchmark areas, then 25% of the costs of that activity could be allocated to each of those four areas. The second approach involved NK and MH reviewing each study's costed activities and assigning a plausible allocation of costs across the Benchmark areas linked to each activity, based on the descriptions offered by included studies. For example, an activity mapped to two Benchmark areas might have 70% of its costs allocated to one (e.g. Surveillance) and the remaining 30% to the other (e.g. Zoonotic disease), based on a study's description of methods and findings. We then added up the costs mapped to each Benchmark area across all activities and summarised our findings for each study.

### Role of the funding source

No additional funding was provided for the conduction of this study. All authors had access to all the data in the study, had final responsibility for the decision to submit for publication prior to submission, and take full responsibility for the content of the article.

## Results

We identified 2220 records from databases. After duplicate removal, we screened the titles and abstracts of 2139 records. From these we assessed 16 full-text documents and finally included three studies.^21^^,^^22^^,^^23^ We identified an additional 24 documents for full-text screening through hand searching of websites, citation searching, and communications with experts/organisations working in this topic area. From these, we identified seven studies that met our final inclusion criteria.^24^, ^25^, ^26^, ^27^, ^28^, ^29^^-^^30^ In total, we included ten studies.^21^, ^22^, ^23^, ^24^, ^25^, ^26^, ^27^, ^28^, ^29^^-^^30^

**Figure 1:**
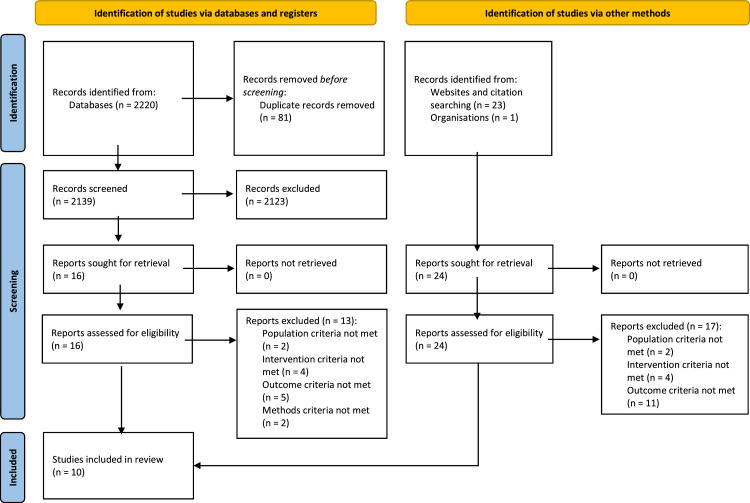
PRISMA Flow Diagram. Figure 1 is based on PRISMA 2020 flow diagram template for systematic reviews and indicates this review's process for identifying, screening, and selecting study records.

All studies were English language studies. Figure 1 summarises our search and screening activities. See appendix for further details.

### Study characteristics

Of the ten included studies, four were publications from peer-reviewed academic journals and six were grey literature reports. Six studies were published during or after 2016,^21^^,^^23^^,^^25^^,^^26^^,^^28^^,^^30^ including three since the onset of the COVID-19 pandemic,^23^^,^^25^^,^^26^ and four were published between 2008 and 2016.^22^^,^^24^^,^^27^^,^^29^ Most studies were produced by international organisations or organisations/authors based in high-income countries. The exceptions to this were one study with three authors primarily affiliated to organisations based in Brazil, China, and Kenya^23^ and one study with an author primarily affiliated to an organisation based in Liberia.^21^

The scope of costed activities varied across the ten included studies (Table 1). Five studies, all published since 2019, described improvements to IHR capacities as some or all of their costs for national-level improvements to preparedness.^21^^,^^25^^,^^28^^,^^30^^,^^26^ Two of these studies based their cost estimates on country-specific needs as measured through the IHR-Monitoring and Evaluation Framework (MEF) assessment tools, such as the Joint External Evaluation (JEE) and IHR State Party Self-Assessment Annual Report (SPAR) processes.^21^^,^^25^ Another two of these studies used results from costed NAPHS; one created an unweighted average of per capita spending based on costed NAPHS and the other extrapolated results from costed NAPHS over a larger group of countries, using unstated methods.^28^^,^^30^ One of these five studies also incorporated the costs of common goods for health.^21^ In the other five studies, all but one published in 2016 or before, authors focused on national-level improvements around the animal-human health interface and zoonoses—using conceptual frameworks such as “One Health”, which complements key aspects of improving IHR capacities.^22^, ^23^, ^24^^,^^27^^,^^29^ One Health also featured in the five studies describing improved IHR capacities – with zoonoses explicitly addressed via technical area #4 Zoonotic disease in the WHO Benchmarks for IHR Capacities.^11^

**Table 1:** Study Methods.

Study	Scope of activities	Global-level activities	National-level activities
Dobson et al. (2020)^23^	The study focused on how to reduce the risks and impact of zoonotic viruses and disease spillover events. • Activities included: wildlife trading monitoring, reducing spillovers from wildlife/livestock, providing early detection and control, reducing deforestation, and ending wild meat trade.	Yes	Yes
FAO et al. (2008)^24^	The study focused on how to diminish the risk and minimise the global impact of epidemics and pandemics due to emerging infections diseases, through the implementation of “*A Strategic Framework for Reducing Risks of Infectious Diseases at the Animal–Human–Ecosystems Interface*”. • Activities included: public health services, veterinary services, wildlife monitoring, communication and social mobilization, international organizations/ participation in regional and global initiatives, and research. • Considered country-specific needs based on Integrated National Action Programs for addressing avian and human influenza.	Yes	Yes (Country-Specific)
Georgetown University CGHSS et al. (2021)^25^	The study focused on addressing gaps in global health security based on the framework and benchmarks provided by the World Health Organization International Health Regulations Monitoring Framework. • Activities covered progress towards “demonstrated capacity” as specified by the benchmarks identified by the Joint External Evaluation (JEE) process; these included policy and coordination, regulations and legislation, information collection and research, veterinary services, biosafety and biosecurity, and communication and population services. • Considered country-specific needs based on most recent available Electronic State Parties Self-Assessment Annual Reporting Tool (e-SPAR) reports.	No	Yes (Country-Specific)
McKinsey & Company (2021)^26^	The study focused on making an economic case for investments in infectious-disease surveillance and preparedness and project requirements to prevent future pandemics. • Activities included: building "always on" response systems, strengthening mechanisms for detecting infectious diseases, integrating efforts to prevent outbreaks, developing healthcare systems that can handle surges while maintaining the provision of essential services, and accelerating R&D for diagnostics, therapeutics, and vaccines.	Yes	Yes
National Research Council (2016)^27^	The study focused on building a framework for global health security through stronger preparedness and response to infectious disease threats. • Activities included: national public health capabilities and infrastructure, international leadership and coordination for preparedness and response, and research and development in the infectious disease arena.	Yes	Yes
Peters (2019)^21^	The study focused on managing the health risks of emergencies and disasters, including epidemics. • Activities included: policy and coordination; regulations and legislation; information collection, analysis and research; communications and persuasion; population services. Incorporated the common goods for health approach. • Considered country-specific needs based on country data collected by the IHR Secretariat at WHO.	No	Yes (Country-Specific)
Pike (2014)^22^	The study focused on analysing the optimal timing for implementation of a globally coordinated adaptive strategy to address a pandemic threat. • Activities included: “business-as-usual” adaptive strategies and the costs of extensive avian mitigation strategies to contain pandemic risk, based on costs of an extensive avian influenza mitigation strategy, expanded to all zoonoses and extrapolated globally.	Yes	Yes
Talisuna (2019)^28^	The study summarised potential pandemic preparedness costs based on 24 national action plans for health security.	No	Yes
World Bank (2012)^29^	The study focused on estimating costs of main prevention and control tasks in public, veterinary, and wildlife health service for all LMICs. • Activities included: public health services, wildlife services, and planning and communication.	No	Yes
World Bank (2019)^30^	The study summarised the potential cost of strengthening preparedness at the country level through implementing national action plans for health security and improving IHR core capabilities.	No	Yes

“Country-specific” is tagged to studies which appeared to rely on some kind of specific national-level assessment that reflected country-specific needs to inform their cost estimates, for all countries assessed in the study. It does not refer to studies that extrapolated costs based on national-level assessments of a select number of countries.

As shown in Table 2, five studies focused on the costs of national-level activities towards improving preparedness.^21^^,^^25^^,^^28^, ^29^, ^30^ The other five studies estimated costs of both national and global level activities.^22^, ^23^, ^24^^,^^26^^,^^27^ Of the ten studies, three estimated the costs of national-level activities across all countries.^22^^,^^25^^,^^26^ The other seven estimated national-level costs for a select group of countries. Three of these seven estimated the costs of improving preparedness for 139 low- and middle-income countries (LMICs),^24^^,^^27^^,^^29^ one for 67 LMICs (representing 95% of the population across all LMICs),^21^ one for the 47 countries of the WHO African region,^28^ one for 31 LMICs that the United States Agency for International Development (USAID) funded “PREDICT” project had previously supported,^23^ and one for 22 LMICs with a costed NAPHS.^30^

**Table 2:** Study Results.

Study	Costing time horizon	National-level activities^A^	Global-level activities^B^	Total costs (baseline year, currency) (bn)	Total costs (2021 US$, bn)	*Costs per capita/year (2021 US$)*
Dobson et al. (2020)	Annual	31 LMICs	Yes	22 - 31·2 (2020, US$)	22·4 - 31·7	*2·92 - 4·14*^C^
FAO et al. (2008)	12 years	139 LMICs	Yes	16·1 (2008, US$)	19·8	*0·29*^C^
		43 LICs	Yes	10·2	12·5	*2·08*
Georgetown University CGHSS et al. (2021)	5 years	All countries^D^		124 (2021, US$)	124	*3·23*^C^
		All LICs		45·9	45·9	*13·73*
		All LMICs^E^		52·5	52·5	*3·60*
		All UMICs		14·6	14·6	*1·02*
		All HICs		10·9	10·9	*1·76*
McKinsey & Company (2021)	10 years	All countries^F^	Yes	285 – 430 (2021, US$)	285 – 430	*3·71 - 5·6*^C^
National Research Council (2016)	Annual	139 LMICs	Yes	4·5 (2016, US$)^G^	4·9	*0·66*^C^
Peters et al. (2019)	Annual	67 LMICs		26·5 (2014, US$)	29·1	*5·23*
		29 LICs		4·9	5·5	*9·47*
		19 LMICs^E^		15	16·7	*6·09*
		19 UMICs		6·2	6·9	*3·08*
Pike et al. (2014)	27 years^H^	All countries^F^	Yes	37·4 – 38·9 (2014, US$)	41·7 – 43·4	*0·21 – 0·22*
Talisuna et al. (2019)	3 years	47 countries, WHO African Region		9 – 10 (2019, US$)^G^	9·3 – 10·3	*2·57 - 3·6*
World Bank (2012)	Annual	139 LMICs		3·4 (2012, US$)^G^	2·1 – 3·9	*0·37 – 0·67*^C^
World Bank (2019)	Annual	22 LMICs		11·2^C^(2019, US$)^G^	11·5	*1·74*

A Numbers of countries noted as in each income status (e.g. UMIC) presented as reported in included studies, country income status may have changed since study publication. As of July 2021, the World Bank classified 27 as economies “low-income”, 55 as “lower-middle income”, 55 as “upper-middle-income”, 80 economies as “high-income”. The World Bank notes that “the term country, used interchangeably with economy, does not imply political independence but refers to any territory for which authorities report separate social or economic statistics”.^1^

B As defined by the 2013 Lancet Commission on Investing in Health, global level costs to support “global functions” that go beyond the boundaries of individual nations to address transnational issues.

C Estimated based on population of studied/referred to countries, based on “National Level Activities”/”Global Level Activities” and population as reported by World Bank (2019) Population Statistics.

D Specified as “196 States Parties to IHR”, study did not further specify the number of countries in each income group.

E LMIC acronym here refers to “Lower Middle Income Countries”, rather than “Low- and Middle-Income Countries”.

F Study did not further specify the number of countries.

G Currency year assumed, not specified by study.

H Estimated based on real options modelling as an optimal timeline. Details of alternate estimates in Appendix.

At a global level, costed activities included contributions to global/regional coordination through international organisations,^22^, ^23^, ^24^^,^^27^^,^^26^ support for research activities, specific R&D for health technologies,^24^^,^^27^^,^^26^ and global networks for manufacturing and distributing vaccines.^26^ At a national level, one study explicitly included the costs of strengthening health systems in the context of preparedness^26^ and two others specified the costs of strengthening key health systems building blocks, such as human resources and, more specifically, investing in health workers.^21^^,^^25^

Only one study of the ten reported separate estimates of capital costs and recurrent costs.^21^ Two other studies described higher costs in the earlier years of their time horizons; though this was attributable to an overall acceleration in preparedness activities, including potential upfront capital costs.^25^^,^^26^

Data sources varied across included studies. Five studies drew upon data from surveys, national-level assessments, and project consultations conducted by country-level stakeholders and/or international organisations.^21^^,^^24^^,^^28^, ^29^, ^30^ For example, two of the five studies relied upon costed NAPHS to create their estimates.^28^^,^^30^ One used government budget data.^22^ Of the other five studies, four relied on a combination of secondary cost data available from peer-reviewed studies and grey literature reports, assumptions from authors and primary data collected through expert interviews.^23^^,^^25^, ^26^, ^27^

Time horizons varied across included studies (Table 2). Five studies only presented estimates of what the annual costs of improvements to preparedness might be.^21^^,^^23^^,^^27^^,^^29^^,^^30^ Four studies estimated costs over 3 year,^28^ 5 year,^25^ 10 year,^26^ and 12 year time horizons.^24^ One study focused on the optimal timeline for investments in preparedness – based on potential expected losses from a pandemic. This equated to 27 years in their final estimates.^22^

Three studies were published following the onset of the COVID-19 pandemic. Two explicitly developed their cost estimates based on challenges identified as a result of the COVID-19 pandemic.^23^^,^^26^ The third focused on existing known gaps in global health security, based on country capacities as reported by the IHR-MEF assessment tools.^25^

Our comparative assessment focuses on included studies' approaches to estimating preparedness costs and, as outlined, does not include a comparative quality assessment. The ten studies did have significant differences in their approaches. These differences are reflected in the variability in their reported methods and results. Studies ranged from offering averages of existing country-level estimates and dedicating only a couple of sentences to describe their costing methods and/or estimated costs, to presenting input-based approaches which built up their cost estimates by considering factors including country specific needs and activities, and complemented their estimates with in-depth descriptions and appendices.^21^, ^26^, ^28^ No study reported the full information required to understand specifically what activities would take place, where they would occur, and how much they would cost.

### Study results

Included studies reported their estimates of preparedness costs in several ways, which - with some adjustment – can be collated for comparison. Two studies covering all countries, both published in 2021, estimated annual costs at US$24·8 billion and between US$28·5 and US$43 billion per year (see Table 2).^25^^,^^26^ The third study covering all countries, published in 2014 and focused more narrowly on zoonoses and the animal-human interface, estimated costs of up to US$1·6 billion per year (US$ 2021 Q1).^22^ Studies with estimates for costs across 139 LMICs ranged from US$1·6 billion to US$4·9 billion per year, which varied as greater costs were added for national- and global-level activities.^24^^,^^27^^,^^29^

Of the other four studies, one estimated costs of up to US$31·7 billion per year. It linked over 90% of these costs to reducing deforestation and the wild meat trade in specific countries. The other three studies differed in their scope of activities and countries covered. They estimated annual costs ranging from US$3 billion to US$15·4 billion per year (US$29·1 billion including upfront capital costs); the studies’ differed in their scope of activities and countries covered.^21^^,^^23^^,^^28^^,^^30^ Table 2 reports range and midpoint estimates where available.

In studies reporting costs by country income status, low-income countries (LICs) had higher total expected costs (Table 2). For example, in the study by Peters and colleagues, per capita annual costs for LICs were US$9·47 compared to US$6·09 in lower-middle income countries and US$3·08 in upper-middle income countries.^21^ However, that study's breakdown of capital and recurrent costs revealed that frontloaded capital investments made up much of that difference in cost estimates. This trend reversed when considering recurrent costs. The association between lower current capacity, lower income countries, and greater costs and improvement needs was also noted by the McKinsey & Company study.^26^ Differences in calculation methods, and the characteristics of countries accounted for, preclude like–for–like comparisons of the absolute costs or per-capita costs estimated across included studies.

One study provided a breakdown of capital and recurrent costs, estimating capital costs of US$13·7 billion and recurrent costs of US$15·4 billion, when considering a single year of improvements.^21^ Two studies provided a breakdown of costs over several years. Both highlighted that costs would be highest in the first year of implementation.^25^^,^^26^ See appendix for further details.

### Study results – mapping activities across the WHO Benchmarks for IHR Capacities

Three of the ten studies only provided top-line aggregated results. Amongst the other seven, there were 59 reported costed activities. These ranged from specific actions (e.g. “start-up costs for national and intermediate laboratory facilities”) to broader categories (e.g. “Population Services”).^21^^,^^23^, ^24^, ^25^, ^26^, ^27^^,^^29^ The number of specific costed activities per study ranged from three to 20. The median number was six.^29^^,^^26^ We included these seven studies in our mapping of costed activities to technical areas in the WHO Benchmarks for IHR Capacities.

Based on our mapping exercise and analysis, we found that costed activities covered 15 of the 18 Benchmark areas. There were some complementary activities as well, such as R&D and health systems strengthening. The three benchmark areas not mapped with any of the seven studies’ costed activities were #13 Linking public health and security authorities, #17 Chemical events and #18 Radiation emergencies. Nevertheless, studies that based estimates on JEE or NAPHS will reflect the costs of these Benchmark areas in their total cost estimates, even if not specified in study reporting.

Table 3 summarises findings from our mapping exercise and our exploratory analysis. The estimated distribution of study costs across the 18 Benchmark areas varied somewhat between our 1st approach and 2nd approach. Yet in several studies, the largest cost drivers appeared to be the same across the two approaches, based on the focus of studies and their reporting. When excluding costs for specified national and global activities which fell outside of the benchmarks (e.g. R&D and health systems costs), we found that the Benchmark areas making up a substantial proportion of costs were often #4 Zoonotic disease and #10 Human resources, and to a lesser extent towards #7 National laboratory system and #9 Surveillance. According to our 2nd approach, #4 Zoonotic disease was the largest cost area in three studies,^23^^,^^24^^,^^29^ #10 Human resources was the largest in two,^21^^,^^25^ and #6 Immunization and R&D for health technologies were the largest in one each.^26^^,^^27^ See appendix for further details.

**Table 3:** Mapping and exploratory analysis of cost drivers (Top 3) across included studies.

	Total number of benchmark areas covered (out of 18)	Major cost drivers (1: Even distribution)	Major cost drivers (2: Author allocation)
**Dobson et al. (2020)**	13	i) National legislation, policy, and financing - 27% ii) Zoonotic disease – 27% iii) Emergency preparedness – 26%	i) Zoonotic disease - 43% ii) Emergency preparedness – 25% iii) Food safety - 16%
**FAO et al. (2008)**	11, also included Research/R&D for health technologies	i) Zoonotic disease - 49% ii) National legislation, policy, and financing - 13% iii) IHR coordination, communication and advocacy and reporting – 13%	i) Zoonotic disease – 49% ii) National legislation, policy, and financing - 19% iii) Risk communication - 8%
**Georgetown University CGHSS et al. (2021)**	6 (18*)	i) Human resources - 34% ii) National laboratory system - 18% iii) Medical countermeasures and personnel deployment - 16%	i) Human resources - 47% ii) *Not specified* (Other) - 23% iii) National laboratory system - 16%
**McKinsey & Company (2021)**	14, also included Research/R&D for health technologies; Health system strengthening.	i) Immunization – 16% ii) Medical countermeasures and personnel deployment - 11% iii) National laboratory system, AMR - 7% (equal)	i) Immunization – 16% ii) Surveillance – 16% iii) Medical countermeasures and personnel deployment - 12%
**National Research Council (2016)**	12, also included Research/R&D for health technologies.	i) Research/R&D for health technologies - 22% ii) National legislation, policy, and financing - 10% iii) *Several areas -* 6%	i) Research/R&D for health technologies - 22% ii) Zoonotic disease - 17% iii) Human resources - 9%
**Peters (2019)**	11 (18*)	i) Human resources – 25% ii) Surveillance – 17% iii) *Several areas –* 6%	i) Human resources – 28% ii) Surveillance – 14% iii) Medical countermeasures and personnel deployment – 11%
**Pike (2014)**	n/a	n/a	n/a
**Talisuna (2019)**	n/a (18*)	n/a	n/a
**World Bank (2012)**	12	i) AMR, National laboratory system, Biosafety and biosecurity, and Surveillance – All 12%	i) Zoonotic disease – 23% ii) National laboratory system – 12% iii) Human resources – 12%
**World Bank (2019)**	n/a (18*)	n/a	n/a

Percentages refer to the proportion of total costs in a study to which each Benchmark area was mapped.

⁎ Some costs not specified in available documentation. Authors noted that they followed WHO IHR-MEF input data, so can be assumed that most/all areas are covered. ** “n/a” (not applicable) used for studies that did not provide a breakdown of costs across activities and were not mapped.

## Discussion

Given that health emergencies can spread rapidly across communities, borders, and regions, identifying the costs of improving health emergency preparedness across multiple countries is important. This systematic review summarises and analyses ten studies that have done just this. It offers a foundation for further work in this area in the health sector and across other sectors.

There was considerable variance in scope of activities considered and calculation methods used in these included studies. More recent studies tended to take a broader perspective than earlier assessments, which centred on epidemic/pandemic preparedness and the animal-human health interface. The scale and scope of these costs offers a useful indication of what could be included in future cost estimates – at the country and global level.

Our mapping exercise and analysis of the distribution of costed activities across Benchmark areas indicated that, across included studies, a large amount of activities (and costs) were often linked to: addressing zoonotic diseases, workforce needs, and broader activities such as R&D for technologies and health systems strengthening. Nevertheless, improvements to health security preparedness require comprehensive investments across all of the Benchmark areas. These findings, and others about variability across studies, should be considered with the conclusions of research by Lee and colleagues in mind: that the potential variability in estimates can be due to different costing approaches being used for improvements in health security preparedness.^31^

Expected costs appeared to be higher in lower income countries in the studies that reported this information; the reason being higher estimated needs for capacity improvements, particularly those involving capital investments.^21^ It is important to note here also that such national-level activities are key contributors to global health security.^20^ Due to study heterogeneity in methods and reporting, including limited accounting for country-specific needs, we did not offer comparisons of per capita cost estimates and could not offer a generalisable unit cost for preparedness in a single technical area or across all aspects of preparedness.

There were some common limitations across included studies. First, studies often reported summary information and had limited in-depth reporting of the inputs and methods used to estimate costs. As a result, it is difficult to assess the reliability of cost estimates. Second, some studies did not specify a conceptual framework for how costs aligned with, or directly contributed to, improvements in preparedness. Third, most studies often did not specify whether costs were contributing to national-level activities or to global-level activities, again we note the key contributions of national-level activities to global health security.^20^ Fourth, at the time of writing, complete information was not available on all included studies. This meant that some costs were classified as “not specified” in our analysis.

Our review also has some limitations. First, we restricted searches to a few databases and the first 1000 results from Google Scholar. Second, searches were in English; though we did not place language restrictions at the time of title and abstract screening. This search strategy may have missed some potentially relevant studies, but we expect that the most recent studies and those informed by current gaps revealed by COVID-19 have likely been captured. Third, groupings of countries based on income status have evolved over time, so there may be minor inaccuracies in our findings across income groups if looking at estimates from today's perspective. Fourth, although our approach to mapping costed activities across the WHO Benchmarks for IHR Capacities is based on information available from studies, it may contain some subjectivity and should be interpreted with caution. Nevertheless, as a first attempt to map the literature on preparedness costing, our exploratory analysis offers some insights into what studies reported to have focused on using a common analytical framework. Moreover, study reporting of methods and specification of activities was sometimes limited – which may have influenced the feasibility and accuracy of mapping costs to Benchmark areas.

This review focused on studies which estimated costs across ten or more countries and at least two technical areas from the from the WHO Benchmarks for IHR Capacities. This meant excluding publicly available estimates for single-countries or single-technical areas. An evidence base which, based on our searches and our knowledge, has not been systematically reviewed. We welcome further analysis in this area.

Future efforts may also focus more on assessing the quality or risk of bias of the evidence base. We note our approach here was in line with a recent systematic review assessing both costing and cost-effectiveness studies for malaria interventions.^32^ The authors concluded that it was inappropriate to use such tools, which might not be fit for purpose for reasons including potential issues of external validity and that studies which estimated only costs (and not consequences) scored lower.

Placed in the context of the potential economic impacts of health emergencies, the costs of implementing the activities suggested by included studies could offer a good return on investment. This includes the upper-bound largest estimates of up to US$ 43 billion per year across all countries, over 10 years. Achieving that good return on investment involves assuming such activities could successfully avert or minimise the expected, and recently observed, losses to health (i.e. illness and death) and economic welfare (i.e. employment and GDP) associated with health emergencies.^1^ The COVID-19 pandemic has led to unprecedented and ever-growing impacts, with trillions of dollars in costs to countries and tens of millions of people being pushed into poverty.^2^^,^^34^ Prior economic studies of health emergencies linked to international disease outbreaks estimated annual economic costs, in addition to harm to people's health and the loss of many lives, of US$ 570 billion from influenza pandemics (expected losses), US$ 53 billion from filoviruses, coronaviruses and other diseases (expected losses), and US$ 6·7 billion from six major outbreaks between 1997 and 2009 (historical estimate).^1^^,^^5^^,^^29^^,^^34^

The scale of the costs in reviewed studies shows the need for substantial and consistent financing going towards health emergency preparedness, including country-level mechanisms and supra-national regional and global ones.^35^^,^^36^ Many preparedness financing mechanisms have been geared towards response, but funding needs to be put in place for preparedness before the onset of health emergencies.^37^^,^^38^ At the global level, much more effort is needed in the financing of common goods for health and the coordination of these efforts. National-level financing is also key. It should be based on assessments of current preparedness activities, updating of national preparedness plans, and integration with broader national health policies, strategies, or plans.^39^

More work can be done to inform future estimates and accelerate efforts to estimate the costs of resources already invested, or planned to be invested, in preparedness at the national level.^31^^,^^40^^,^^41^ This can include further specifying the global cost estimates collated as part of recent and ongoing efforts to advocate for improved pandemic preparedness, which already account for the findings of some studies included in this review.^2^ Assessments of the role of cross-sectoral activities and investments would also be useful.

Across global, regional, and national levels, there is also an opportunity and need to be more explicit about what represents better value for the available financing. Such analysis could draw upon evidence about the efficiency, effectiveness, and distributional impacts of activities. It could also comprehensively account for experiences with managing the risks and impacts of COVID-19. This could help to prioritise activities like those recommended by recent and ongoing international initiatives to direct immediate increases in support for preparedness worldwide.^42^ The case for financing of preparedness and improving resilience is clear. Specific investments today can respond to future challenges to health security.^43^, ^44^, ^45^ Neglecting such investments ignores the likelihood of another pandemic on the scale of COVID-19, or even greater.

This review poses important policy and research questions. The accepted assumption is that health emergency preparedness is typically underfunded. We need to know more about what areas are underfunded, to better target funding and understand the potential impacts of insufficient investment. We need to understand how the cost estimates of included studies, and similar studies focused on specific countries and specific aspects of preparedness, fit into strategic planning and the prioritisation of activities in health systems and beyond. More can be done to catalogue and assess studies with more specific levels of focus (i.e. number of countries, technical areas), like those we identified during study record screening, but did not meet the inclusion criteria here – typically due to only focusing on one benchmark area, such as #4 Zoonotic disease or #6 Immunization.^46^, ^47^, ^48^ From our searches, we note one example of such a study that estimated costs across two countries.^49^ Most had a single country and single area focus.^50^^,^^51^ Other global level studies and investment cases, such as those focused on polio risk management, may also offer insights.^52^^,^^53^

This review can inform future costing methods and the creation of country-specific cost estimates to inform planning and priority setting by policymakers and stakeholders. Improved guidance could help with interpretation and comparison across studies/countries. As could greater transparency and detail in the reporting of methods by studies focused on this topic.

Future work should also be informed, where possible, by research on Benchmark areas at the single country level, or on specific programs which have been implemented across several countries.^54–56^ Such work may include the development of costing resources and price tags for preparedness across countries, all with the aim of contributing to a greater understanding of what investments are needed, where they are needed, and how to sustainably fund them.

## Declaration of interests

All authors work as salaried staff/consultants for the World Health Organization (Geneva, Switzerland).

## Appendix Supplementary materials

Image, application 1

## Footnotes

1 World Bank (2021) World Bank Country and Lending Groups. Available from https://datahelpdesk.worldbank.org/knowledgebase/articles/906519..
