# Supplementary material for: The costs of improving health emergency preparedness: A systematic review and analysis of multi-country studies
Source: eClinicalMedicine. 2022 Jan 27;44:101269. doi: 10.1016/j.eclinm.2021.101269 (PMC8802087; doi:10.1016/j.eclinm.2021.101269)
Supplement: Supplementary file 1 [file mmc1.docx]

Appendix File for “The costs of improving health emergency preparedness: a systematic review and analysis of multi-country studies”

Table of Contents

[Appendix 1 - Search Strategy 1](#_Toc90459228)

[Appendix 2 – Definitions 2](#_Toc90459239)

[Appendix 3 – Study Inclusion and Full-Text Screening 4](#_Toc90459240)

[Appendix 4 – WHO Benchmarks for International Health Regulations (IHR) (2015) Capacities (2019) 9](#_Toc90459241)

[Appendix 5 – Data Collection Form 13](#_Toc90459242)

[Appendix 6 - Updates to PROSPERO Protocol 14](#_Toc90459243)

[Appendix 7 - Study Characteristics (Authorship) 14](#_Toc90459244)

[Appendix 8 – Study Results (Summary) 16](#_Toc90459245)

[Appendix 9 – Study Results - Preparedness cost drivers 21](#_Toc90459246)

# Appendix 1 - Search Strategy

## Databases (Exportable)

### PubMed

1. Pandemic*[ti] OR "emergency response"[tiab] OR “health emergenc*”[tiab] OR epidemic*[ti] OR outbreak*[tiab] OR disaster*[tiab] OR "health systems"[tiab] OR "health capacity"[tiab] OR "global rapid response"[tiab] OR "disease intelligence"[tiab] OR "biosecurity"[tiab] OR "emerging disease"[tiab] OR "bioterrorism"[tiab] OR "Zoonotic disease"[tiab] OR "emerging infectious disease"[tiab] OR "animal disease"[tiab] OR “health security”[tiab]
2. Preparedness[tiab] OR preparation[tiab] OR Capacity[tiab] OR readiness[tiab] OR Vigilance[tiab] OR Mitigation[tiab] OR strengthen*[tiab]OR capabilit*[tiab] OR surveillance[tiab] OR "prevention"[tiab] OR "one health"[tiab]
3. Cost[ti] OR costs[ti] OR USD[tiab] OR price[ti] OR financing[ti] OR expense[ti] OR economic*[ti]
4. "EDRM"[tiab] OR "emergency and disaster risk management"[tiab]
5. (#1 AND #2 AND #3) OR #4
6. Limit to publications after 2000/1/1

### Web of Science

1. pandemic OR epidemic OR "health emergenc*" OR outbreak  OR “health security”
2. prevention OR preparedness OR mitigation
3. cost OR costing OR costs OR USD
4. #1 AND #2 AND #3
5. Refined by: [excluding] Databases: (MEDLINE) AND RESEARCH DOMAINS: (SOCIAL SCIENCES)
6. Timespan: 2000-2021. Databases:  WOS, BCI, KJD, MEDLINE, RSCI, SCIELO, ZOOREC.
7. Search language=Auto

## National Health Service Economic Evaluation Database (NHS EEDS)

- Pandemic OR epidemic OR "health emergenc*" OR outbreak OR “health security”) AND (prevention OR preparedness OR mitigation) AND (cost OR costing OR costs OR USD)

### EconLit

- (pandemic OR epidemic OR "health emergenc*" OR outbreak OR “health security”) AND (prevention OR preparedness OR mitigation) AND (cost OR costing OR costs OR USD)

### Google Scholar

- (pandemic OR epidemic OR "health emergency" OR outbreak) AND (prevention OR preparedness OR mitigation) AND (cost OR costing OR costs OR USD)

## Databases (Handsearching)

### Prevention Web ([https://www.preventionweb.net](https://www.preventionweb.net/))

- Separate searches:
  - Epidemic AND Preparedness AND Cost.
  - Pandemic AND Preparedness AND Cost.

### World Bank Open Knowledge Repository (<https://openknowledge.worldbank.org/>)

- Pandemic OR epidemic OR "health emergenc*" OR outbreak OR “health security”) AND (prevention OR preparedness OR mitigation) AND (cost OR costing OR costs OR USD).

## Websites (Handsearching)

- GPMB (<https://apps.who.int/gpmb/annual_report.html>)
- Independent Panel for Pandemic Preparedness and Response (<https://theindependentpanel.org/>)
- One Health Commission (<https://www.onehealthcommission.org/en/resources__services/one_health_library/>)
- Resolve to Save Lives (<https://preventepidemics.org/>)
- High Level Independent Panel for Financing the Global Commons for Pandemic Preparedness and Response (<https://pandemic-financing.org/>)

# Appendix 2 – Definitions

Our manuscript adheres to definitions specified by WHO in relation to health emergency preparedness.^[[1]](#footnote-2)^ For example:

- An emergency is “*an event or imminent threat that produces or has the potential to produce a range of health consequences, and which requires coordinated action, usually urgent and often non-routine, and may pose a substantial risk of significant morbidity or mortality in a community*”
- Emergency preparedness relates to “*the knowledge and capacities developed by governments, response and recovery organizations, communities and individuals to effectively anticipate, respond to and recover from the impacts of likely, imminent or current emergencies”.*

# Appendix 3 – Study Inclusion and Full-Text Screening

The below table summarises our inclusion criteria in the PICOS format (Population, Intervention, Comparison, Outcome, Study design). These criteria ensured that we captured studies covering a wide range of preparedness activities that attempted to understand the resource needs across many countries.

| **Population** | Activities conducted at the global level and/or at the national level across at least ten countries. |
| --- | --- |
| **Intervention** | Cover two or more of the 18 technical areas of the WHO Benchmarks for International Health Regulations (IHR) Capacities and did not focus only animal health or those that only focused on activities that occurred during the response or recovery phases of health emergencies. |
| **Comparison** | *No restrictions*. |
| **Outcome** | Costs based on market-traded inputs (which have an observable market price). |
| **Study design** | *No restrictions*. |

The below table summarises the sources used and decisions made during our full-text screening of candidate studies in our systematic review.

|  | Study | Decision and Reason | Source |
| --- | --- | --- | --- |
| 1 | Alleweld et al (2021) Economic evaluation of whole genome sequencing for pathogen identification and surveillance - results of case studies in Europe and the Americas 2016 to 2019. | Exclude – Population | Databases |
| 2 | Alleweldt et al (2012) Cost of national prevention systems for animal diseases and zoonoses in developing and transition countries | Exclude – Intervention | Databases |
| 3 | Beach et al (2007) Farm economics of bird flu | Exclude – Outcome | Hand-searching/  Snowball searching |
| 4 | Berry et al (2018) The Economic Case for a Pandemic Fund | Exclude – Outcome | Databases |
| 5 | Carias et al (2016) Preventive malaria treatment for contacts of patients with Ebola virus disease in the context of the west Africa 2014–15 Ebola virus disease response: an economic analysis | Exclude – Population | Databases |
| 6 | Carrasco et al (2011) Strategies for antiviral stockpiling for future influenza pandemics: a global epidemic-economic perspective | Exclude – Intervention | Hand-searching/  Snowball searching |
| 7 | Commission on a Global Health Risk Framework for the Future (2016) The Neglected Dimension of Global Security - A Framework to Counter Infectious Disease Crises. | Include | Hand-searching/  Snowball searching |
| 8 | Council on Foreign Relations (2020) Improving Pandemic Preparedness - Lessons From COVID-19 | Exclude – Population | Hand-searching/  Snowball searching |
| 9 | CSIS Commission on Strengthening America’s Health Security (2019) Ending the Cycle of Crisis and Complacency in U.S. Global Health Security A Report of the CSIS Commission on Strengthening America’s Health Security | Exclude – Population | Hand-searching/  Snowball searching |
| 10 | Dobson et al (2020) Ecology and economics for pandemic prevention. | Include | Databases |
| 11 | FAO et al (2008) Contributing to One World, One Health - A Strategic Framework for Reducing Risks of Infectious Diseases at the Animal–Human–Ecosystems Interface. | Include | Hand-searching/  Snowball searching |
| 12 | Gouglas et al (2018) Estimating the cost of vaccine development against epidemic infectious diseases: a cost minimisation study | Exclude – Intervention | Databases |
| 13 | Georgetown University Center for Global Health Science and Security and Talus Analytics (2021) Estimating cost requirements to build global health security capacity. | Include | Hand-searching/  Snowball searching |
| 14 | Harvard Global Helath Institute (2018) Global Monitoring Of Disease Outbreak Preparedness - Preventing The Next Pandemic A Shared Framework | Exclude – Outcome | Hand-searching/  Snowball searching |
| 15 | Häsler et al (2013) The economic value of One Health in relation to the mitigation of zoonotic disease risks. | Exclude – Outcome | Databases |
| 16 | IPBES (2020) Workshop Report on Biodiversity and Pandemics of the Intergovernmental Platform on Biodiversity and Ecosystem Services | Exclude – Outcome | Hand-searching/  Snowball searching |
| 17 | Jonas (2013) Pandemic Risk | Exclude – Outcome | Hand-searching/  Snowball searching |
| 18 | Katz et al (2018) Strengthening health security: an intuitive and user-friendly tool to estimate country-level costs | Exclude – Outcome | Databases |
| 19 | Kraus et al (2020) ﻿Measuring development assistance for health systems strengthening and health security: an analysis using the Creditor Reporting System database. | Exclude – Outcome | Hand-searching/  Snowball searching |
| 20 | Lugnér et al (2012) Cost effectiveness of vaccination against pandemic influenza in European countries: mathematical modelling analysis | Exclude – Intervention | Hand-searching/  Snowball searching |
| 21 | Madhav et al (2017) Pandemics: risks, impacts, and mitigation | Exclude – Outcome | Databases |
| 22 | McKinsey & Company (2021) Not the last pandemic: Investing now to reimagine public-health systems. | Include | Hand-searching/  Snowball searching |
| 23 | McLeod et al (2007) Economic issues in vaccination against highly pathogenic avian influenza in developing countries. | Exclude – Intervention | Databases |
| 24 | Medema et al (2004) Modeling pandemic preparedness scenarios: health economic implications of enhanced pandemic vaccine supply | Exclude – Intervention | Databases |
| 25 | Moon and Vaidya (2018) Investing for a rainy day challenges in financing national preparedness for outbreaks | Exclude – Outcome | Hand-searching/  Snowball searching |
| 26 | NTI et al (2020) Concept Note - Global Health Security Challenge Fund | Exclude – Outcome | Hand-searching/  Snowball searching |
| 27 | OIE (2007) Prevention and control of animal diseases worldwide Economic analysis – Prevention versus outbreak costs - Final Report - Part 1 | Exclude – Intervention | Hand-searching/  Snowball searching |
| 28 | Peters et al (2019) Financing Common Goods for Health: Core Government Functions in Health Emergency and Disaster Risk Management. | Include | Databases |
| 29 | Pike et al (2014) Economic optimization of a global strategy to address the pandemic threat. | Include | Databases |
| 30 | Radin and Eleftheriades (2021) Financing Pandemic Preparedness and Response - Background Paper 14, Commissioned by the Independent Panel for Pandemic Preparedness and Response | Exclude – Outcome | Hand-searching/  Snowball searching |
| 31 | Schar et al (2018) A framework for stimulating economic investments to prevent emerging diseases. | Exclude – Outcome | Hand-searching/  Snowball searching |
| 32 | Schmets et al (2017) Interconnectedness of UHC and health security: First Face-to-Face Meeting of the UHC2030 Working Group on Support to Countries with Fragile or Challenging Operating Environments. | Exclude – Methods  (Included Peters et al, 2019 to account for final calculations) | Databases |
| 33 | Stenberg et al (2017) Financing transformative health systems towards achievement of the health Sustainable Development Goals: a model for projected resource needs in 67 low-income and middle-income countries | Exclude – Methods  (Included Peters et al, 2019 to account for final calculations) | Databases |
| 34 | Talisuna et al (2019) Joint external evaluation of the International Health Regulation (2005) capacities - current status and lessons learnt in the WHO African region. | Include | Hand-searching/  Snowball searching |
| 35 | WHO (2006) Global Pandemic influenza Action Plan to Increase Vaccine Supply. | Exclude – Intervention | Hand-searching/  Snowball searching |
| 36 | World Bank (2012) People, Pathogens and Our Planet : The Economics of One Health | Include | Hand-searching/  Snowball searching |
| 37 | World Bank (2019) Pandemic Preparedness Financing - Status Update | Include | Hand-searching/  Snowball searching |
| 38 | World Bank, International Working Group on Financing Preparedness (2017) From Panic and Neglect to Investing in Health Security - Financing Pandemic Preparedness at a National Level | Exclude – Outcome | Hand-searching/  Snowball searching |
| 39 | Yamey et al (2017) Financing of international collective action for epidemic and pandemic preparedness | Exclude – Outcome | Databases |
| 40 | Yamey et al (2019) Financing Global Common Goods for Health - When the World is a Country | Exclude – Outcome | Hand-searching/  Snowball searching |

# Appendix 4 – WHO Benchmarks for International Health Regulations (IHR) Capacities (2019)

The below table details descriptions and objectives across the WHO Benchmarks for International Health Regulations (IHR) Capacities, as published in 2019.^[[2]](#footnote-3)^

| **#** | **Technical area** | **#** | **Description and Objectives** |
| --- | --- | --- | --- |
| 1 | National legislation, policy, and financing | 1.1 | Domestic legislation, laws, regulations, policy and administrative requirements are available in all relevant sectors and effectively enable compliance with the IHR. ***Objective****: To assess, adjust and align domestic legislation, laws, regulations, policy and administrative requirements in all relevant sectors to enable compliance with the IHR.* |
|  |  | 1.2 | Financing is available for the implementation of IHR capacities. ***Objective****: To ensure financing is available for the implementation of IHR capacities.* |
|  |  | 1.3 | Financing available for timely response to public health emergencies. ***Objective****: To develop a financing mechanism to ensure that funds are available for timely response to public health emergencies.* |
| 2 | IHR coordination, communication and advocacy and reporting | 2.1 | The IHR NFP is fully functional. ***Objective****: To establish a fully functional IHR NFP.* |
|  |  | 2.2 | Multisectoral IHR coordination mechanism effectively supports the implementation of prevention, detection and response activities. ***Objective****: To establish a multisectoral IHR coordination mechanism to support the implementation of prevention, detection and response activities.* |
| 3 | Antimicrobial resistance (AMR) | 3.1 | Effective multisectoral coordination on AMR. ***Objective****: To develop and implement a multisectoral national action plan on AMR.* |
|  |  | 3.2 | Surveillance system of AMR is in place. ***Objective****: To develop a national AMR surveillance system that integrates surveillance of AMR in pathogens of concern to human and animal health and agriculture.* |
|  |  | 3.3 | Infection prevention and control is in place. ***Objective****: To develop a functioning infection prevention and control system for healthcare facilities and farms.* |
|  |  | 3.4 | Optimize use of antimicrobial medicines in human and animal health and agriculture. ***Objective****: To ensure appropriate use of all antimicrobials in human and animal health and agriculture.* |
| 4 | Zoonotic disease | 4.1 | Coordinated surveillance system is in place for priority zoonotic diseases/pathogens. ***Objective****: Strengthen coordinated surveillance systems for priority zoonotic diseases/path.* |
|  |  | 4.2 | Functional mechanism to respond to priority zoonotic diseases in place. ***Objective****: Strengthen mechanism to respond to zoonotic diseases.* |
| 5 | Food safety | 5.1 | Surveillance systems in place for the detection and monitoring of foodborne diseases and food contamination. ***Objective****: Strengthen surveillance systems for foodborne diseases and food contamination.* |
|  |  | 5.2 | A functional mechanism is in place for the response and management of food safety emergencies. ***Objective****: Strengthen mechanisms for response and management of food safety emergencies.* |
| 6 | Immunization | 6.1 | Optimum vaccine coverage (measles) as part of a national programme. ***Objective****: Increase vaccine coverage for priority vaccine preventable diseases in the country.* |
|  |  | 6.2 | Provision of national vaccine access and delivery. ***Objective****: Strengthening capacity for vaccine access and delivery to target population.* |
| 7 | National laboratory system | 7.1 | Laboratory testing for detection of priority diseases is in place. ***Objective****: Strengthening laboratory testing for detection of priority diseases.* |
|  |  | 7.2 | Specimen referral and transport system are in place for all relevant sectors. ***Objective****: Strengthen specimen referral and transport system.* |
|  |  | 7.3 | Effective national diagnostic network is in place. ***Objective****: Establish effective national diagnostic network.* |
| 8 | Biosafety and biosecurity | 8.1 | Whole-of-government biosafety and biosecurity system is in place for all sectors (including human, animal (domestic animals and wildlife) and environment facilities). ***Objective****: To develop and implement a biosafety and biosecurity system for all sectors (including human, animal (domestic and wildlife) and environment facilities) to minimize the risk of accidental or intentional infection of laboratory staff or release of hazardous pathogens.* |
|  |  | 8.2 | Biosafety and biosecurity training and practices in all relevant sectors (including human, animal (domestic animals and wildlife) and environment). ***Objective****: To develop a public health workforce that is available and trained to enable early detection, prevention, preparedness and response to potential events of international concern at all levels of health systems to effectively implement IHR.* |
| 9 | Surveillance | 9.1 | Functional surveillance system to identify potential events of concern for public health and health security is in place. ***Objective****: Strengthen surveillance system.* |
|  |  | 9.2 | Surveillance system is supported by electronic tools. ***Objective****: Application of electronic tools for surveillance system.* |
|  |  | 9.3 | Systematic analysis of surveillance data for action is in place.  ***Objective****: Conduct analysis of data for action* |
| 10 | Human resources | 10.1 | An up-to-date, multisectoral workforce strategy is in place. ***Objective****: To develop and implement an up-to-date workforce strategy for a functional multisectoral health workforce.* |
|  |  | 10.2 | Human resources are available to effectively implement IHR. ***Objective****: To develop a public health workforce that is available and trained to prevent, detect, assess, notify, report and respond to public health risks and acute events of domestic and international concern and health service provision (i.e. epidemic preparedness and control) at all levels of health systems to effectively implement IHR.* |
|  |  | 10.3 | In-service trainings are available. ***Objective****: To develop a functioning and accredited continuing professional education programme through in-service trainings at national and subnational levels.* |
|  |  | 10.4 | ﻿Field epidemiology training programme or other applied epidemiology training programme is in place  ***Objective****: To establish a sustainable field epidemiology training programme or other applied epidemiology training programme* |
| 11 | Emergency preparedness | 11.1 | Strategic emergency risk assessments conducted, and emergency resources identified, mapped and utilized. ***Objective****: To assess and assign priorities to risks based on analyses of hazards exposures and vulnerabilities and capacities, and develop inventories and maps of available resources for emergency preparedness and response..* |
|  |  | 11.2 | Multisectoral planning for health emergency preparedness and response is in place. ***Objective****: Development and implementation of multisectoral and multihazard emergency preparedness measures including emergency response plans.* |
| 12 | Emergency response operations | 12.1 | Functional emergency response coordination is in place. ***Objective****: To develop coordination mechanisms for emergency response.* |
|  |  | 12.2 | Emergency operations centre (EOC) capacities, procedures and plans are in place. ***Objective****: Develop emergency operation capacities.* |
|  |  | 12.3 | Emergency exercise management programme is in place. ***Objective****: Develop the emergency exercise management programme.* |
| 13 | Linking public health and security authorities | 13.1 | Public health and security authorities (law enforcement, border control, customs) linked during a suspect or confirmed biological, chemical or radiological event. ***Objective****: Strengthening the linkage between public health and security authorities during potential events of national concern.* |
| 14 | Medical countermeasures and personnel deployment | 14.1 | System is in place for activating and coordinating medical countermeasures during a public health emergency. ***Objective****: To develop a functional system for activating and coordinating health personnel during a public health emergency.* |
|  |  | 14.2 | System is in place for activating and coordinating health personnel during a public health emergency. ***Objective****: To develop a functional system for activating and coordinating health personnel during a public health emergency.* |
|  |  | 14.3 | Case management procedures implemented for relevant IHR hazards. ***Objective****: Develop and implement case management procedures for all relevant IHR hazards.* |
| 15 | Risk communication | 15.1 | Risk communication systems for unusual events and emergencies is in place. ***Objective****: To develop a system for risk communication for unusual events and emergencies.* |
|  |  | 15.2 | Coordination of risk communication is effective. ***Objective****: Strengthen coordination for risk communication.* |
|  |  | 15.3 | Effective communication with communities. ***Objective****: Strengthen communication engagement with communities.* |
| 16 | Points of entry | 16.1 | Routine capacities at points of entry are in place. ***Objective****: Establishment of routine capacities at designated points of entry.* |
|  |  | 16.2 | Effective public health response at points of entry. ***Objective****: Strengthen capacity for effective public health response at points of entry.* |
| 17 | Chemical events | 17.1 | Mechanisms are in place for surveillance, alert and response to chemical events or emergencies. ***Objective****: Establish policies, legislation, plans and capacities for surveillance, alert and response to chemical events or emergencies.* |
| 18 | Radiation emergencies | 18.1 | Mechanism is in place for detecting and responding to radiological and nuclear emergencies. ***Objective****: Establish a mechanism to detect and respond to radiological and nuclear emergencies.* |

# Appendix 5 – Data Collection Form

We collected data using a standard form in Microsoft Excel, that drew on the CHEERS statement and a previous review of the costs and benefits of interventions aimed at major infectious disease threats.(1,2)

Our data collection form focused on study characteristics, including study details (title, author, date of publication), study methods (research question, hazard, geographical setting, time horizon, currency conversion and inflation adjustment methods, costing perspective, costing method and data sources) and study results (total cost estimates and cost drivers data).

Once this information was collected, we then used the form to collect more detailed information about study methods and results, including preparedness related activities, costed items, costing methods, data sources, currency information, and cost estimate for each activity.

The below table displays our data collection form.

| **Item** | **Study Information** |
| --- | --- |
| **A.   Study Info** |  |
| i.  Study - e.g *World Bank (2012) People, Pathogens and Our Planet - The Economics of One Health* |  |
| ii. Review Author Conducting Extraction |  |
| iii. Date  of extraction |  |
| **B.   Study Methods** |  |
| i. Primary Objective/Research question |  |
| ii. Hazard  - Specify in terms used by paper, e.g. Pandemics, Epidemics  - Note what the explicit and implicit focus are (implicit focus may be indicated by the estimated benefits of improved preparedness, such as pandemic prevention) |  |
| iii. Geographical scope of costing |  |
| iv. Intervention(s)/Activity(ies) |  |
| v. Costing perspective |  |
| vi. Calculation methods/Costing approach  - General approach - Bottom-up/top-down (e.g. Ingredients costing, strategic/localised approaches)  - Specific methods - Price x Quantity, regression adjustment,  - Account for benefits approach, because it reflects the costing approach too. |  |
| vii. Time horizon |  |
| viii. Currency, year, and currency conversion methods (if used) |  |
| ix. Discounting/Inflation Adjustment |  |
| x. Uncertainty (e.g. role in methods, how accounted for) |  |
| xi. Data source   (e.g. primary data, secondary/published data) |  |
| **C.    Study Results** |  |
| i. Findings (Summary) |  |
| ii. Findings (Detailed) |  |
| iii. Cost Drivers |  |
| iv. Uncertainty in findings   (e.g. drives and sources of uncertainty - inputs, model assumptions, etc.) |  |
| v. Heterogeneity   in findings (e.g. differences between subgroups – focus on described differences as detailed findings will provide also) |  |
| **D.   Study Discussion** |  |
| i. Contributions to policy/research (*reported by study authors*) |  |
| ii. Limitations   (*reported by study authors*) |  |

# Appendix 6 - Updates to PROSPERO Protocol

We note the following updates to PROSPERO CRD42021254428, as highlighted in the main manuscript:

*Inclusion Criteria*

Initially, we considered including studies that focused on at least two or more countries and at least one technical area, but concluded that included studies needed to account for multiple technical areas and several country settings.

The purpose of this review was to inform, from a health sector standpoint, the development of national- and global-level targets for investments in preparedness, encompassing a range of activities, to offer a regional and global perspective on addressing epidemic and pandemic risks. These perspectives are critical given how interconnected and complementary preparedness capacities are between countries.

As a result, it became clear following submission of our protocol to PROSPERO that our focus should be on studies offering analysis across both multiple technical areas and groups of countries – using, as a reference point, that the smallest WHO region – by country size – covers 11 countries (WHO SEARO). Moreover, accounting for multiple technical areas ensured that at least one area focused mainly on health sector aspects; noting the critical role of the animal health sector for activities under Technical Area #4 “Zoonotic disease” in the WHO Benchmarks for IHR Capacities.

# Appendix 7 - Study Characteristics (Authorship)

The below table described the geographic spread of study authorship, based on the locations of the primary organisations to which authors were affiliated at time of publication or the location of the organisations producing grey literature reports.

| Study | Geographic spread of study authorship, based on affiliations |
| --- | --- |
| Dobson et al (2020) | Most authors (12/15) were primarily affiliated with organisations based in the United States of America (USA). |
| FAO et al (2008) | Report published by variety of international organisations. |
| Georgetown University CGHSS et al (2021) | Organisation based in the USA. |
| McKinsey & Company (2021) | Organisation based in the USA. |
| National Research Council (2016) | Organisation based in the USA. |
| Peters et al (2019) | Authors were primarily affiliated to organisations/international organisations based in the USA, United Kingdom, Switzerland, and Liberia. |
| Pike et al (2014) | Authors were primarily affiliated to organisations based in the USA. |
| Talisuna et al (2019) | Authors were primarily affiliated to international organisations (WHO Regional Office for Africa and WHO Headquarters). |
| World Bank (2012) | Report published by an international organisation. |
| World Bank (2019) | Report published by an international organisation. |

# Appendix 8 – Study Results (Summary)

The below table displays “Gross Domestic Product, Deflator, Seasonally Adjusted, Index” data from the IMF which was used to inflate the costs of included studies. Data here was taken from that for the United States of America, as all included studies were reported in USD (across various years).^[[3]](#footnote-4)^ The earliest included year is 2008, in line with the publication year of the earliest study included in this review. Estimates from studies were all reported in USD. Nevertheless, we acknowledge this inflation approach has limitations because US inflation rates may not accurately reflect price changes in many low- and middle-income countries, particularly for local non-tradable resources (e.g. workforce).^[[4]](#footnote-5)^

The base years and currencies for cost data were obtained from studies; if the studies did not report the base year for their cost estimates, we instead used the year when the study was published.

| Indicator Code | 2008 | 2009 | 2010 | 2011 | 2012 | 2013 | 2014 | 2015 | 2016 | 2017 | 2018 | 2019 | 2020 | Q1 2021 |
| --- | --- | --- | --- | --- | --- | --- | --- | --- | --- | --- | --- | --- | --- | --- |
| Source Data | 98·1 | 98·8 | 100·0 | 102·1 | 104·0 | 105·9 | 107·8 | 108·9 | 110·0 | 112·1 | 114·8 | 116·8 | 118·2 | 120·26 |
| Inflator - 2021 USD (Q1) | 1·226 | 1·217 | 1·203 | 1·178 | 1·156 | 1·136 | 1·115 | 1·105 | 1·093 | 1·073 | 1·048 | 1·030 | 1·017 | 1·000 |

The below table summarises overall study results, including figures presented as totals and sub-totals. In this table, we round the values for absolute costs to five significant figures.

| Study | Total/ Sub-Total | Scope | Baseline Costs - Currency (Year) | Baseline Costs - Total | Inflator - 2021 USD (Q1) | Costs – Total  (2021 Q1 USD) | Costs - Per Year  (2021 Q1 USD) | Costs - Per Capita/Year  (2021 Q1 USD) |
| --- | --- | --- | --- | --- | --- | --- | --- | --- |
| Dobson et al (2020) | Total  (Low-end scenario) | - Activities: Global and National - Population: 7,673,656,870 (World Bank (2019) Population - Global, 2019) - Time Horizon: Annual estimate | 2020, USD | 21,993,000,000 | 1·017 | 22,371,000,000 | 22,371,000,000 | 2·92 |
| Dobson et al (2020) | Total  (High-end scenario) | *Same as above, except high-scenario for estimates* | 2020, USD | 31,211,000,000 | 1·017 | 31,748,000,000 | 31,748,000,000 | 4·14 |
| FAO et al (2008) | Total | - Costs: Global and National (139 LMICs) - Population: 5,594,000,000 (World Bank (2019) Population - Low & middle income, 2008) - Time Horizon: 12 years (2008-2020) | 2008, USD | 10,228,000,000 | 1·226 | 12,538,000,000 | 1,045,000,000 | 2·08 |
| FAO et al (2008) | Sub-Total  (LICs only) | - Costs: Global and National (43 LICs) - Population: 503,324,676 (World Bank (2019) Population - Low income, 2008) - Time Horizon: 12 years (2008-2020) | 2008, USD | 16,116,000,000 | 1·226 | 19,756,000,000 | 1,646,000,000 | 0·29 |
| Georgetown University CGHSS et al (2021) | Total | - Costs: National (196 countries) - Population: 7,673,656,870 (World Bank (2019) Population - Global, 2019) - Time Horizon: 5 years | 2021, USD | 124,000,000,000 | 1·000 | 124,000,000,000 | 24,800,000,000 | 3·23 |
| Georgetown University CGHSS et al (2021) | Sub-Total (LICs only) | - Costs: National (LICs) - Population: 668,454,965 (World Bank (2019) Population - Global, 2019) - Time Horizon: 5 years | 2021, USD | 45,900,000,000 | 1·000 | 45,900,000,000 | 9,180,000,000 | 13·73 |
| Georgetown University CGHSS et al (2021) | Sub-Total (LMICs only) | - Costs: National (LMICs) - Population: 2,913,369,050 (World Bank (2019) Population - Global, 2019) - Time Horizon: 5 years | 2021, USD | 52,500,000,000 | 1·000 | 52,500,000,000 | 10,500,000,000 | 3·60 |
| Georgetown University CGHSS et al (2021) | Sub-Total (UMICs only) | - Costs: National (UMICs) - Population: 2,855,841,760 (World Bank (2019) Population - Global, 2019) - Time Horizon: 5 years | 2021, USD | 14,600,000,000 | 1·000 | 14,600,000,000 | 2,920,000,000 | 1·02 |
| Georgetown University CGHSS et al (2021) | Sub-Total (HICs only) | - Costs: National (HICs) - Population: 1,235,991,090 (World Bank (2019) Population - Global, 2019) - Time Horizon: 5 years | 2021, USD | 10,900,000,000 | 1·000 | 10,900,000,000 | 2,180,000,000 | 1·76 |
| McKinsey & Company (2021) | Total (Lower-end scenario) | - Costs: Global and National (196 countries) - Population: 7,673,656,870 (World Bank (2019) Population - Global, 2019) - Time Horizon: 10 years | 2021, USD | 285,000,000,000 | 1·000 | 285,000,000,000 | 28,500,000,000 | 3·71 |
| McKinsey & Company (2021) | Total (Higher-end scenario) | *Same as above, except higher-end scenario for estimates·* | 2021, USD | 430,000,000,000 | 1·000 | 430,000,000,000 | 43,000,000,000 | 5·60 |
| McKinsey & Company (2021) | Total (Midpoint) | *Same as above, except midpoint for estimates·* | 2021, USD | 356,535,000,000 | 1·000 | 356,535,000,000 | 35,654,000,000 | 4·65 |
| National Research Council (2016) | Total | - Costs: Global and National (139 countries) - Population: 7,424,000,000 (World Bank (2019) Population - Global, 2016) - Time Horizon: Annual | 2016, USD | 4,500,000,000 | 1·093 | 4,920,000,000 | 4,920,000,000 | 0·66 |
| Peters et al (2019) | Total | - Costs: National (67 LMICs) - Population: 5,646,468,005 (Stenberg et al (2017)) - Time Horizon: Annual | 2014, USD | 26,050,000,000 | 1·115 | 29,052,000,000 | 29,052,000,000 | 5·23 |
| Peters et al (2019) | Total (29 LICs only) | - Costs: National (29 LICs) - Population: 587,214,594 (Stenberg et al (2017)) - Time Horizon: Annual | 2014, USD | 4,900,000,000 | 1·115 | 5,465,000,000 | 5,465,000,000 | 9·47 |
| Peters et al (2019) | Total (29 LICs only) | - Costs: National (19 LMICs) - Population: 2,783,503,308 (Stenberg et al (2017)) - Time Horizon: Annual | 2014, USD | 15,000,000,000 | 1·115 | 16,728,000,000 | 16,728,000,000 | 6·09 |
| Peters et al (2019) | Total (19 UMICs only) | - Costs: National (19 LMICs) - Population: 2,275,750,103 (Stenberg et al (2017)) - Time Horizon: Annual | 2014, USD | 6,200,000,000 | 1·115 | 6,914,000,000 | 6,914,000,000 | 3·08 |
| Peters et al (2019) | Sub-Total (Capital Costs) | - Costs: National (67 LMICs) - Population: 5,646,468,005 (Stenberg et al (2017)) - Time Horizon: Annual | 2014, USD | 12,300,000,000 | 1·115 | 13,717,000,000 | 13,717,000,000 | 2·46 |
| Peters et al (2019) | Sub-Total (Capital Costs, 29 LICs only) | - Costs: Capital Costs (29 LICs) - Population: 587,214,594 (Stenberg et al (2017)) - Time Horizon: Annual | 2014, USD | 2,500,000,000 | 1·115 | 2,788,000,000 | 2,788,000,000 | 4·83 |
| Peters et al (2019) | Sub-Total (Capital Costs, 29 LICs only) | - Costs: Capital Costs (19 LMICs) - Population: 2,783,503,308 (Stenberg et al (2017)) - Time Horizon: Annual | 2014, USD | 6,800,000,000 | 1·115 | 7,584,000,000 | 7,584,000,000 | 2·75 |
| Peters et al (2019) | Sub-Total (Capital Costs, 19 UMICs only) | - Costs: Capital Costs (19 LMICs) - Population: 2,275,750,103 (Stenberg et al (2017)) - Time Horizon: Annual | 2014, USD | 3,000,000,000 | 1·115 | 3,346,000,000 | 3,346,000,000 | 1·51 |
| Peters et al (2019) | Sub-Total (Recurrent Costs) | - Costs: Recurrent Costs (67 LMICs) - Population: 5,646,468,005 (Stenberg et al (2017)) - Time Horizon: Annual | 2014, USD | 13,800,000,000 | 1·115 | 15,390,000,000 | 15,390,000,000 | 2·77 |
| Peters et al (2019) | Sub-Total (Recurrent Costs, 29 LICs only) | - Costs: Recurrent Costs (29 LICs) - Population: 587,214,594 (Stenberg et al (2017)) - Time Horizon: Annual | 2014, USD | 2,400,000,000 | 1·115 | 2,677,000,000 | 2,677,000,000 | 0·18 |
| Peters et al (2019) | Sub-Total (Recurrent Costs, 29 LICs only) | - Costs: Recurrent Costs (19 LMICs) - Population: 2,783,503,308 (Stenberg et al (2017)) - Time Horizon: Annual | 2014, USD | 8,200,000,000 | 1·115 | 9,145,000,000 | 9,145,000,000 | 3·33 |
| Peters et al (2019) | Sub-Total (Recurrent Costs, 19 UMICs only) | - Costs: Recurrent Costs (19 LMICs) - Population: 2,275,750,103 (Stenberg et al (2017)) - Time Horizon: Annual | 2014, USD | 3,200,000,000 | 1·115 | 3,569,000,000 | 3,569,000,000 | 1·57 |
| Pike et al (2014) | Total (Lower-end scenario - Mitigation)^[[5]](#footnote-6)^ | - Costs: Global and National - Population: 7,254,000,000 (World Bank (2019) Population - Low & middle income, 2014) - Time Horizon: 27 years (Economic optimization approach) | 2014, USD | 37,400,000,000 | 1·115 | 41,709,000,000 | 1,545,000,000 | 0·21 |
| Pike et al (2014) | Total (Higher-end scenario - Mitigation) | - Costs: Global and National - Population: 7,254,000,000 (World Bank (2019) Population - Low & middle income, 2014) - Time Horizon: 27 years (Economic optimization approach) | 2014, USD | 38,900,000,000 | 1·115 | 43,382,000,000 | 1,607,000,000 | 0·22 |
| Talisuna et al (2019) | Total (Lower-end scenario) | - Costs: National (47 countries, WHO African Region) - Population: N/A (Estimates based on reported per capita expenditure) - Time Horizon: 3 years | 2019, USD | 9,000,000,000 | 1·030 | 9,266,000,000 | 3,089,000,000 | 2·57* |
| Talisuna et al (2019) | Total (Higher-end scenario) | - Costs: National (47 countries, WHO African Region) - Population: N/A (Estimates based on reported per capita expenditure) - Time Horizon: 3 years | 2019, USD | 10,000,000,000 | 1·030 | 10,295,000,000 | 3,432,000,000 | 3·60* |
| World Bank (2012) | Total (Lower-end scenario) | - Costs: National (139 countries) - Population: 5,894,000,000 (World Bank (2019) Population - Low & middle income, 2012) - Time Horizon: Annual - Scenario: Risk of disease prevalence is low· | 2012, USD | 1,900,000,000 | 1·156 | 2,196,000,000 | 2,196,000,000 | 0·37 |
| World Bank (2012) | Total (Higher-end scenario) | - Costs: National (139 countries) - Population: 5,894,000,000 (World Bank (2019) Population - Low & middle income, 2012) - Time Horizon: Annual - Scenario: Risk of disease prevalence is high | 2012, USD | 3,400,000,000 | 1·156 | 3,930,000,000 | 3,930,000,000 | 0·67 |
| World Bank (2019) | Total | - Costs: National (Based on 22 countries) - Population: 6,437,665,780 (World Bank (2019) Population - Low & middle income, 2019) - Time Horizon: Annual | 2019, USD (not specified) | 11,201,538,457  ^[[6]](#footnote-7)^ | 1·030 | 11,532,000,000 | 11,532,000,000 | 1·74 |

# Appendix 9 – Study Results - Preparedness cost drivers

The below tables and graphs summarise data from our mapping and exploratory analysis of cost drivers across included studies.

The first table is keys for subsequent tables.

| # | Study |
| --- | --- |
| 1 | Dobson et al (2020) |
| 2 | FAO et al (2008) |
| 3 | Georgetown University CGHSS et al (2021) |
| 4 | McKinsey & Company (2021) |
| 5 | National Research Council (2016) |
| 6 | Peters et al (2019) |
| 7 | Pike et al (2014) |
| 8 | Talisuna et al (2019) |
| 9 | World Bank (2012) |
| 10 | World Bank (2019) |

We used each study’s descriptions of methods and results to map all costed activities to one or more of the 18 areas of the WHO Benchmarks for IHR (2005) Capacities.^[[7]](#footnote-8)^ LC and EP conducted the initial mapping during data collection and then NK and MH, experts in the implementation of preparedness capacities at national- and global-levels, reviewed and updated the mapping of costed activities.

The below table displays a summary of which Benchmark areas appeared in each study and the number of times each Benchmark area appeared across all of a study’s costed activities.

| Study | # |
| --- | --- |
| Dobson et al (2020) | 13 Benchmark areas (1,2,3,4,5,6,7,8,9,10,11,12,15). |
| FAO et al (2008) | 11 Benchmark areas (1,2,4,7,8,9,10,11,12,15,16), additional areas included Research/R&D for health technologies |
| Georgetown University CGHSS et al (2021) | 6 Benchmark areas (4,7,9,10,14,16), some costs not specified in available documentation. Authors noted that they followed JEE/IHR, so can be assumed that most/all areas covered. |
| McKinsey & Company (2021) | 14 Benchmark areas (1,3,4,5,6,7,8,9,10,11,12,14,15,16), additional areas included Research/R&D for health technologies; Health system strengthening. |
| National Research Council (2016) | 12 Benchmark areas (1, 2,3,4,6,7,8,9,10,11,12,15) |
| Peters et al (2019) | 11 Benchmark areas (1,2,3,7,9,10,11,12,14,15,16). Authors noted that they followed JEE/IHR, so can be assumed that most/all areas covered. |
| Pike et al (2014) | N/A (No activities specified, study provides only total/aggregate estimate of costs) |
| Talisuna et al (2019) | N/A (No activities specified, study provides only total/aggregate estimate of costs) |
| World Bank (2012) | 1,2,3,4,6,7,8,9,10,11,12,15 |
| World Bank (2019) | N/A (No activities specified, study provides only total/aggregate estimate of costs) |

The below table summarises the findings from our mapping of the types of activities that were costed in included studies. Based on our mapping exercise and analysis, we found that, across all studies, costed activities covered 15 of the 18 Benchmark areas and some complementary activities as well (e.g. R&D and health systems strengthening). The three benchmark areas not identified across studies’ costed activities were #13 Linking public health and security authorities, #17 Chemical events and #18 Radiation emergencies. However, studies that based estimates on JEE or NAPHS will reflect the costs of these Benchmark areas in their total cost estimates, even if not specified in study reporting.

| Study | 1 | 2 | 3 | 4 | 5 | 6 | 7 | 8 | 9 | 10 |
| --- | --- | --- | --- | --- | --- | --- | --- | --- | --- | --- |
| 1) National legislation, policy, and financing | Yes | Yes | - | Yes | Yes | Yes | - | - | Yes | - |
| 2) IHR coordination, communication and advocacy and reporting | Yes | Yes | - | - | Yes | Yes | - | - | Yes | - |
| 3) Antimicrobial resistance (AMR) | Yes | - | - | Yes | Yes | Yes | - | - | Yes | - |
| 4) Zoonotic disease | Yes | Yes | Yes | Yes | Yes | - | - | - | Yes | - |
| 5) Food safety | Yes | - | - | Yes | - | - | - | - | - | - |
| 6) Immunization | Yes | - | - | Yes | Yes | - | - | - | Yes | - |
| 7) National laboratory system | Yes | Yes | Yes | Yes | Yes | Yes | - | - | Yes | - |
| 8) Biosafety and biosecurity | Yes | Yes | - | Yes | Yes | - | - | - | Yes | - |
| 9) Surveillance | Yes | Yes | Yes | Yes | Yes | Yes | - | - | Yes | - |
| 10) Human resources | Yes | Yes | Yes | Yes | Yes | Yes | - | - | Yes | - |
| 11) Emergency preparedness | Yes | Yes | - | Yes | Yes | Yes | - | - | Yes | - |
| 12) Emergency response operations | Yes | Yes | - | Yes | Yes | Yes | - | - | Yes | - |
| 13) Linking public health and security authorities | - | - | - | - | - | - | - | - | - | - |
| 14) Medical countermeasures and personnel deployment | - | - | Yes | Yes | - | Yes | - | - | - | - |
| 15) Risk communication | Yes | Yes | - | Yes | Yes | Yes | - | - | Yes | - |
| 16) Points of entry | - | Yes | Yes | Yes | - | Yes | - | - | - | - |
| 17) Chemical events | - | - | - | - | - | - | - | - | - | - |
| 18) Radiation emergencies | - | - | - | - | - | - | - | - | - | - |
| Other - Research | - | Yes | - | - | - | - | - | - | - | - |
| Other - Health Systems Strengthening | - | - | - | Yes | - | - | - | - | - | - |
| Other - Health Technologies - R&D | - | - | - | Yes | Yes | - | - | - | - | - |
| No activities specified | - | - | Yes | - | - | - | Yes | Yes | - | Yes |

We used the aforementioned mapping to conduct an exploratory analysis of how costs were distributed across the 18 Benchmark areas in and between included studies, using two separate approaches. The first approach involved assuming that if a costed activity was mapped to two or more Benchmark areas, then the estimated costs of that activity were evenly distributed across those Benchmark areas. For example, if an activity was mapped to four Benchmark areas, then 25% of the costs of that activity could be allocated to each of those four Benchmark areas. We then added up costs allocated to each Benchmark area for each study (across all activities) and summarised our findings for comparison.

The table below displays the results of this analysis using our first approach (the “Even Distribution” approach). Percentages are presented in decimals out of 100 and colour scales are used to highlight areas associated with a higher percentage of costs.

| **Study** | **1** | **2** | **3** | **4** | **5** | **6** | **7** | **8** | **9** | **10** |
| --- | --- | --- | --- | --- | --- | --- | --- | --- | --- | --- |
| 1) National legislation, policy, and financing | 0·27 | 0·13 | - | 0·03 | 0·1 | 0·026 | - | - | 0·08 | - |
| 2) IHR coordination, communication and advocacy and reporting | 0·01 | 0·13 | - | - | 0·06 | 0·026 | - | - | 0·08 | - |
| 3) Antimicrobial resistance (AMR) | 0 | - | - | 0·07 | 0·06 | 0·093 | - | - | 0·12 | - |
| 4) Zoonotic disease | 0·27 | 0·49 | 0·01 | 0·04 | 0·06 | - | - | - | 0·08 | - |
| 5) Food safety | 0·16 | - | - | 0·02 | - | - | - | - | - | - |
| 6) Immunization | 0 | - | - | 0·16 | 0·06 | - | - | - | 0·05 | - |
| 7) National laboratory system | 0·01 | 0·02 | 0·18 | 0·07 | 0·06 | 0·093 | - | - | 0·12 | - |
| 8) Biosafety and biosecurity | 0 | 0·02 | - | 0·02 | 0·06 | - | - | - | 0·12 | - |
| 9) Surveillance | 0·01 | 0·02 | 0·04 | 0·2 | 0·06 | 0·17 | - | - | 0·12 | - |
| 10) Human resources | 0 | 0·02 | 0·34 | 0·02 | 0·06 | 0·25 | - | - | 0·08 | - |
| 11) Emergency preparedness | 0·26 | 0·02 | - | 0·03 | 0·06 | 0·08 | - | - | 0·04 | - |
| 12) Emergency response operations | 0 | 0·02 | - | 0·04 | 0·06 | 0·03 | - | - | 0·05 | - |
| 13) Linking public health and security authorities | - | - | - | - | - | - | - | - | - | - |
| 14) Medical countermeasures and personnel deployment | - | - | 0·16 | 0·11 | - | 0·09 | - | - | - | - |
| 15) Risk communication | 0 | 0·09 | - | 0·03 | 0·06 | 0·05 | - | - | 0·08 | - |
| 16) Points of entry | - | 0·02 | 0·03 | 0·04 | - | 0·09 | - | - | - | - |
| 17) Chemical events | - | - | - | - | - | - | - | - | - | - |
| 18) Radiation emergencies | - | - | - | - | - | - | - | - | - | - |
| Other - Research | - | 0·03 | - | - | - | - | - | - | - | - |
| Other - Health Systems Strengthening | - | - | - | 0·05 | - | - | - | - | - | - |
| Other - Health Technologies - R&D | - | - | - | 0·05 | 0·22 | - | - | - | - | - |
| Activities not specified | - | - | 0·23 | - | - | - | 1 | 1 | - | 1 |

The second approach involved NK and MH reviewing each study’s costed activities and assigning a plausible allocation of costs across the Benchmark areas linked to each activity, based on descriptive information reported by studies. An activity mapped to two Benchmark areas, for example, might have 70% of the costs might be allocated to one (e.g. Surveillance) and 30% to the other (e.g. Zoonotic Events) based on a study’s description of methods and findings. Using the first and second approaches separately, we then added up costs allocated to each Benchmark area for each study (across all activities) and summarised our findings for comparison. To complement this analysis we summarised factors driving preparedness costs, based on information reported in included studies.

The table below displays the results of this analysis using our second approach (the “Plausible allocation” approach). Percentages are presented in decimals out of 100 and colour scales are used to highlight areas associated with a higher percentage of costs.

| **Study** | **1** | **2** | **3** | **4** | **5** | **6** | **7** | **8** | **9** | **10** |
| --- | --- | --- | --- | --- | --- | --- | --- | --- | --- | --- |
| 1) National legislation, policy, and financing | 0·13 | 0·19 | - | 0·02 | 0·05 | 0·01 | - | - | 0·02 | - |
| 2) Coordination and NFP communications | 0 | 0·04 | - | - | 0·03 | 0·02 | - | - | 0·04 | - |
| 3) Antimicrobial resistance (AMR) | 0 | - | - | 0·09 | 0·04 | 0·1 | - | - | 0·05 | - |
| 4) Zoonotic disease | 0·43 | 0·49 | 0·01 | 0·06 | 0·17 | - | - | - | 0·23 | - |
| 5) Food safety | 0·16 | - | - | 0·02 | - | - | - | - | - | - |
| 6) Immunization | 0 | - | - | 0·16 | 0·03 | - | - | - | 0·04 | - |
| 7) National laboratory system | 0·01 | 0·02 | 0·16 | 0·09 | 0·09 | 0·1 | - | - | 0·12 | - |
| 8) Biosafety and biosecurity | 0 | 0·01 | - | 0·01 | 0·07 | - | - | - | 0·09 | - |
| 9) Surveillance | 0·01 | 0·03 | 0·02 | 0·16 | 0·08 | 0·14 | - | - | 0·11 | - |
| 10) Human resources | 0 | 0·04 | 0·47 | 0·01 | 0·09 | 0·28 | - | - | 0·12 | - |
| 11) Emergency preparedness | 0·25 | 0·02 | - | 0·02 | 0·01 | 0·07 | - | - | 0·02 | - |
| 12) Emergency response operations | 0 | 0·02 | - | 0·05 | 0·05 | 0·03 | - | - | 0·07 | - |
| 13) Linking public health and security authorities | - | - | - | - | - | - | - | - | - | - |
| 14) Medical countermeasures and personnel deployment | - | - | 0·09 | 0·12 | - | 0·11 | - | - | - | - |
| 15) Risk communication | 0 | 0·08 | - | 0·01 | 0·06 | 0·09 | - | - | 0·09 | - |
| 16) Points of entry | - | 0·02 | 0·03 | 0·04 | - | 0·06 | - | - | - | - |
| 17) Chemical events | - | - | - | - | - | - | - | - | - | - |
| 18) Radiation emergencies | - | - | - | - | - | - | - | - | - | - |
| Other - Research | - | 0·03 | - | - | - | - | - | - | - | - |
| Other - Health Systems Strengthening | - | - | - | 0·08 | - | - | - | - | - | - |
| Other - Health Technologies - R&D | - | - | - | 0·05 | 0·22 | - | - | - | - | - |
| Activities not specified | - | - | 0·23 | - | - | - | 1 | 1 | - | 1 |

The distribution of study costs across activity areas varied somewhat between our 1^st^ approach and 2^nd^ approach, though in several cases the major cost drivers remained the same across the two approaches.

When excluding costs for specified national and global preparedness activities which fell outside of the benchmarks (e.g. R&D and health systems costs), we found that, based on the focus of studies and their reporting, that the Benchmark areas making up a substantial proportion of costs were often #4 Zoonotic disease and #10 Human resources, and to a lesser extent towards #7 National laboratory system and #9 Surveillance.

According to our 2^nd^ approach, #4 Zoonotic disease was the largest cost area in three studies (Dobson et al, 2020; FAO et al, 2008; World Bank, 2012), #10 Human resources was the largest in two (CGHSS and Talus Analytics, 2021; Peters et al, 2019), and #6 Immunization and R&D for health technologies were the largest in one each (McKinsey & Company, 2021; NRC, 2016).

1. WHO (2020) Glossary of health emergency and disaster risk management terminology (<https://www.who.int/publications/i/item/glossary-of-health-emergency-and-disaster-risk-management-terminology>) [↑](#footnote-ref-2)
2. WHO (2019) WHO Benchmarks for International Health Regulations (IHR) Capacities. (<https://www.who.int/ihr/publications/9789241515429/en/>) [↑](#footnote-ref-3)
3. IMF (2021) IMF Data - <https://data.imf.org> [↑](#footnote-ref-4)
4. ﻿Turner et al (2019) Adjusting for Inflation and Currency Changes Within Health Economic Studies - https://doi.org/https://doi.org/10.1016/j.jval.2019.03.021 [↑](#footnote-ref-5)
5. Pike et al (2014) also present the costs of an “adaptation policy”, involving four hypothetical policies. However, we do not include these costs here due to the “mitigation policy” being deemed cost effective.

   Options for the “adaptation policy”: “A” increases the current policy spending by a factor of 1.1 ($75.6 billion), option “B” targets increases the current policy spending by a factor of 2.5 ($171.9 billion), option “C” increases the current policy spending by a factor of 5 ($343.7 billion), and option “D” increases the current policy spending by a factor of 10 ($687.5 billion). [↑](#footnote-ref-6)
6. Worked backwards from per capita figure. [↑](#footnote-ref-7)
7. World Health Organization (2019) WHO Benchmarks for IHR (2005) Capacities. [↑](#footnote-ref-8)
